# Overexpression of GDP dissociation inhibitor 1 gene associates with the invasiveness and poor outcomes of colorectal cancer

**DOI:** 10.1080/21655979.2021.1967031

**Published:** 2021-09-13

**Authors:** Xiao Xie, Huajiang Lin, Xiaolei Zhang, Pengtao Song, Xiangyi He, Jing Zhong, Jiemin Shi

**Affiliations:** a Department of Gastroenterology, Huzhou Central Hospital, Affiliated Central Hospital Huzhou University, Affiliated Huzhou Hospital Zhejiang University School of Medicine, Huzhou, Zhejiang Province, China; b Department of Pathology, Huzhou Central Hospital, Affiliated Central Hospital Huzhou University, Affiliated Huzhou Hospital Zhejiang University School of Medicine, Huzhou, Zhejiang Province, China; c Huzhou Key Laboratory of Molecular Medicine, Huzhou Central Hospital, Affiliated Central Hospital Huzhou University, Affiliated Huzhou Hospital Zhejiang University School of Medicine, Huzhou, Zhejiang Province, China

**Keywords:** GDP dissociation inhibitor 1, colorectal cancer, prognostic biomarker

## Abstract

GDP dissociation inhibitor (*GDI)* regulates the GDP/GTP exchange reaction of most Rab proteins by inhibiting GDP dissociation. This study evaluated the potential prognostic and predictive value of *GDI1* in colorectal cancer (CRC). To address the prognostic power of *GDI1*, we performed individual and pooled survival analyses on six independent CRC microarray gene expression datasets. *GDI1*-enriched signatures were also analyzed. Kaplan–Meier and Cox proportional analyses were employed for survival analysis. An immunohistochemistry (IHC) analysis was performed to validate the clinical relevance and prognostic significance of the GDI1 protein level in CRC tissue samples. The results revealed that *GDI1* mRNA level was significantly linked with the aggressiveness of CRC, which is compatible with gene set enrichment analysis. A meta-analysis and pooled analysis demonstrated that a higher mRNA *GDI1* expression was dramatically correlated with a worse survival in a dose-dependent manner in CRC patients. Further IHC analysis validated that the protein expression of GDI1 in both cytoplasm and membrane also significantly impacted the outcome of CRC patients. In CRC patients with stage III, chemotherapy significantly reduced the relative risk of death in low-*GDI1* subgroup (hazard ratio (HR) = 0.22; 95% confidence interval (95% CI) 0.09–0.56, *p* = 0.0003), but not in high-*GDI1* subgroup (HR = 0.63; 95% CI 0.35–1.14, *p* = 0.1137). Therefore, both high mRNA and protein levels of GDI1 were significantly related to poor outcomes in CRC patients. *GD11* may serve as a prognostic biomarker for CRC.

## Introduction

Colorectal cancer (CRC) has the third highest morbidity and the second highest mortality, accounting for 10% of all annually diagnosed cancers and cancer-related deaths both in the world [1]. Genetic and environmental factors play a significant role in the development of CRC [2–4]. The pathogenesis of CRC, either intrinsic or acquired, is multifactorial and multistep [5,6]. The outcome of CRC is associated with the stage at diagnosis, with a 5-year survival rate of 90% at early diagnosis and less than 10% when distant metastases develop [7]. Therefore, it is critical to develop reliable biomarkers with the sound capability of predicting CRC metastasis. Various biomarkers have been proposed to predict CRC outcomes, such as MMP7 [8], miR21, miR106a, miR135, miR17-92 [9,10] CK19, CK20, or Carcinoembryonic antigen (CEA) [11,12] miR29a and miR92a [13], Cancer antigen 19–9 (CA 19–9) [14–18]. However, few have been used to guide clinical CRC treatment [19].

*GDI1*, one subtype of GDP dissociation inhibitors, regulates the GDP/GTP exchange reaction of Rab family. Small GTP-binding proteins of the ras superfamily are involved in the vesicular trafficking of molecules between cellular organelles. Studies have shown that Rab protein’s C-terminal region can bind to Rab GDP dissociation inhibitor (Rab *GDI*) nonspecificly [20]. Meanwhile, Rab protein acts as a molecular switch of vesicle transport, and the key regulator of intracellular vesicle transport [21–24]. It has been reported that Rab protein is closely related to the occurrence and development of some tumors [25–27]. In contrast, Rab *GDI2*, as the other subtype of GDI, is expressed in all cancers, such as prostate cancer [28], human NSCLC [29] and breast cancer McF-7 cells treated with retinoic acid [30]. Highly hemologic to *GDI2, GDI1* gene is located in Xq28, also known as RABGDIA or XAP-4 [31]. Rab *GDI1* is mainly expressed in nervous and sensory tissues. Rab GDI reduces the expression of Rab3A, an active protein related to synaptic vesicles, in the brain tissues of schizophrenic patients [32]. Previous studies have shown that *GDI1* is associated with intellectual disability [33], hyperexcitability [34,35] and memory formation in the forebrain region of mice [36]. However, the role of *GDI1* in tumors has not been reported.

We extracted six CRC datasets from the GEO (Gene Expression Omnibus) and TCGA (The Cancer Genome Atlas). Bioinformatics analysis was conducted on the microarray data of more than 1000 patients with CRC, and 10 related genes were screened out, including *GDI1* that was then selected as the research object. Based on previous findings, we hypothesized that *GDI1* might play an essential role(s) in tumorigenesis, growth, and chemosensitivity of CRC. This study aims to validate if *GDI1* is associated with the aggressiveness and survival of CRC. Our goal is to develop *GDI1* as a prognostic biomarker to predict the outcome of CRC patients.

## Methods

### Gene expression datasets

A total of 6 CRC gene expression datasets containing survival information was downloaded from the Array Express database (www.ebi.ac.uk/arrayexpress), including GSE39582, GSE38832, GSE29623, and GSE28722. Meanwhile, the datasets of TCGA-COAD1 and TCGA-COAD2 were downloaded from The Cancer Genome Atlas (TCGA)(www.cancergenome.nih.gov). These datasets were further used to evaluate the role of *GDI1* in CRC prognosis. Detailed information on the gene expression datasets is summarized in Supplementary Table 1.

### Gene set enrichment analysis

A gene set enrichment analysis (GSEA) was performed on the CRC microarray dataset, according to the protocol on the Broad Institute Gene Set Enrichment Analysis website (www.broad.mit.edu/gsea) or from related references [37,38]. Briefly, datasets and phenotype label files were created and loaded into GSEA software (v2.0.13). The gene sets were downloaded from the Gene Expression Omnibus GEO (http://www.cancergenome.nih.gov/geo). The phenotype labels were set as high-*GDI1* and low-*GDI1* in GSE39582 dataset. The number of permutations was set to 1000. A ranked-list metric was generated based on the signal-to-noise ratio calculated with the mean scaled difference according to the standard deviation (SD).

### Patients

The Institutional Ethics Committees of Affiliated Huzhou Hospital, Zhejiang, approved the use of CRC patients’ data (No.20190517). Informed consent was given to the patients. In total, 133 CRC cases who had undergone surgical operations were collected. Inclusion criteria [39]: (i) primary CRC; (ii) having pathological diagnosis; (iii) being followed up within 5 years. Exclusion criteria: (i) failing to get consent; (ii) lost to follow-up; and (iii) multiple-cancer patients. The distribution of demographic data is described in Table 3. All participants in the ZJU set were Chinese from the eastern part of China (Zhejiang).

**Table 1.** Demographic distribution of *GDI1* in a pooled dataset of colon cancer

	High (%)	Low (%)	P-values*	OS HR (95%CI)**	P-values†
Gender					
Male	288(49.7)	292 (50.3)	0.6531	Reference	
Female	245(51.0)	235(49.0)		0.83(0.68–1.01)	0.0679
Age					
<50 yrs	54(41.5)	76(58.5)	0.0338	Reference	
≥50 yrs	508(51.4)	480(48.6)		1.44(1.03–2.09)	0.0304
AJCC TNM stage					
0-I	52(33.5)	103(66.5)	<0.0001	Reference	
II	268(48.3)	287(51.7)		1.45(0.93–2.40)	0.1022
III	222(54.3)	187(45.7)		2.17(1.38–3.58)	0.0004
IV	105(60.3)	69(39.7)		8.98(5.70–14.9)	<0.0001
Tumor stage					
T1	4(44.4)	5(55.5)		Reference	
T2	28(35.0)	52(65.0)		1.07(0.19–20.0)	0.9481
T3	182(52.7)	163(47.3)	0.0149	2.69(0.60–47.4)	0.2419
T4	35(59.3)	24(40.7)		5.87(1.23–105.0)	0.0214
Lymph node involvement					
N0	128(44.6)	159(55.4)		Reference	
N1	70(57.4)	52(42.6)	0.0072	2.05(1.32–3.17)	0.0015
N2	51(60.7)	33(39.3)		4.31(2.78–6.65)	<0.0001
Metastasis					
M0	148(47.3)	165(52.7)		Reference	
M1	42(68.9)	19(31.1)	0.0018	4.19(2.61–6.62)	<0.0001
Differentiation					
Poor	5(50.0)	5(50.0)	0.5913	Reference	
Mod	26(51.0)	25(49.0)		0.56(0.35–0.95)	0.0334
Well	1(25.0)	3(75.0)		0.46(0.17–1.09)	0.0804
Molecular subtype					
C1	41(35.3)	75(64.7)	<0.0001	Reference	
C2	56(53.9)	48(46.1)		0.72(0.44–1.16)	0.1178
C3	21(28.0)	54(72.0)		0.64(0.35–1.11)	0.1114
C4	46(78.0)	13(22.0)		1.79(1.12–2.84)	0.0162
C5	92(60.5)	60(39.5)		0.85(0.57–1.29)	0.4544
C6	28(46.7)	32(53.3)		1.00(0.59–1.65)	0.9963
MMR status					
dMMR	46(61.3)	29(38.7)	0.0628	Reference	
pMMR	221(49.8)	223(50.2)		1.30(0.82–2.20)	0.2743
Adjuvant chemotherapy					
No	172(50.2)	171(49.8)	0.7236	Reference	0.5785
Yes	132(48.7)	139(51.3)		0.92(0.70–1.22)	

There are 1060, 1118, 1293, 493, 493, 374, 65, 566, 519, 614 cases in gender, age, TNM stage, Tumor Grade, Molecular subtype, MMR status, and adjuvant chemotherapy.

* *p* values were based on the Pearson Chi-square test. † Statistical significance, *P* < 0.05; ‡ Statistical significance, *P* < 0.01.

**Table 2.** Uni- and multivariate analysis for *GDI1* and survival in CRC datasets

Datasets		Overall survival		Disease-free survival	
		HR(95%CI)*	Adjusted HR(95%CI)*	HR(95%CI)*	Adjusted HR(95%CI)*
GSE39582	Q_1_	Reference	Reference	Reference	Reference
	Q_2_	1.22(0.79–1.92)	0.97(0.62–1.53)	1.74(1.09–2.83)†	1.53(0.95–2.51)
	Q_3_	1.72(1.13–2.65)†	1.43(0.94–2.21)	2.20(1.39–3.55)‡	1.76(1.11–2.86)†
	Q_4_	1.82(1.21–2.77)‡	1.34(0.88–2.07)	2.29(1.45–3.68)‡	2.02(1.28–3.27)‡
GSE38832	Q_1_	Reference	Reference	Reference	Reference
	Q_2_	0.78(0.19–2.97)	0.94(0.23–3.63)	1.31(0.22–9.98)	0.67(0.11–5.36)
	Q_3_	0.48(0.12–1.84)	1.53(0.35–6.25)	0.45(0.05–3.88)	0.24(0.03–2.14)
	Q_4_	2.48(0.94–7.70)	1.33(0.51–4.15)	0.89(0.10–7.79)	0.42(0.05–3.83)
GSE29623	Q_1_	Reference	Reference	Reference	Reference
	Q_2_	2.20(0.73–7.29)	1.91(0.60–6.68)	7.01(1.20–132.49)†	4.67(0.76–90.3)
	Q_3_	1.56(0.50–5.31)	1.42(0.42–5.18)	3.27(0.42–66.17)	7.06(0.75–163.7)
	Q_4_	1.25(0.35–4.52)	1.74(0.43–7.14)	2.40(0.23–51.70)	23.3(1.75–616.5)†
GSE28722	Q_1_	Reference	Reference	Reference	Reference
	Q_2_	0.72(0.34–1.53)	0.35(0.15–0.77)†	1.47(0.49–4.86)	0.63(0.19–2.23)
	Q_3_	1.11(0.54–2.29)	0.89(0.42–1.86)	2.73(1.01–8.59)†	2.52(0.91–8.03)
	Q_4_	0.86(0.39–1.83)	0.60(0.27–1.28)	1.82(0.61–6.04)	1.56(0.52–5.19)
TCGA-COAD-1	Q_1_	Reference	Reference	Reference	Reference
	Q_2_	1.90(0.86–4.39)	1.44(0.64–3.39)	1.61(0.80–3.31)	1.35(0.66–2.81)
	Q_3_	1.99(0.94–4.46)	1.31(0.60–3.03)	1.40(0.70–2.88)	1.13(0.55–2.38)
	Q_4_	3.08(1.50–6.79)‡	1.88(0.88–4.30)	1.92(0.97–3.89)	1.61(0.80–3.31)
TCGA-COAD-2	Q_1_	Reference	Reference	Reference	Reference
	Q_2_	2.07(0.65–7.77)	1.58(0.48–6.02)	1.19(0.22–6.46)	1.01(0.18–5.77)
	Q_3_	2.26(0.71–8.47)	1.48(0.43–5.81)	1.57(0.34–7.98)	1.17(0.22–6.90)
	Q_4_	2.35(0.76–8.68)	1.54(0.46–6.02)	2.37(0.62–11.3)	2.02(0.44–11.2)
Pooled set	Q_1_	Reference	Reference	Reference	Reference
	Q_2_	1.26 (0.93–1.71)	1.03 (0.74–1.44)	1.68(1.19–2.39)‡	1.33(0.93–1.93)
	Q_3_	1.46(1.09–1.97)†	1.33(0.97–1.84)	1.84(1.31–2.60)‡	1.57(1.10–2.26)†
	Q_4_	1.78(1.34–2.38)‡	1.35(0.99–1.87)	2.05(1.46–2.90)‡	1.74(1.23–2.50)‡

Uni- and multivariate analysis were conducted to evaluate HR of *GDI1.*

* For multivariate analysis, HR was adjusted by age and stage in GSE39582 and GSE28722 sets; and HR was adjusted by stage in GSE38832, GSE29623, TCGA-COAD-1and TCGA-COAD-2 sets.

† Statistical significance, *p* < 0.05; ‡ Statistical significance, *p* < 0.01.

**Table 3.** Demographic distribution of colorectal cancers from ZJU set

	GDI1 cytoplasm			GDI1 Membrane		
	Low (%)	High (%)	p-value	Low (%)	High (%)	p-value
Age						
<40	5(62.5)	3(37.5)		3(37.5)	5(62.5)	
40–49	5(26.3)	14(73.7)		7(36.8)	12(63.2)	
50–59	14(43.8)	18(56.3)		20(62.5)	12(37.5)	
60–69	18(47.4)	20(52.6)		25(65.8)	13(34.2)	
70–79	16(59.3)	11(40.7)		19(70.4)	8(29.6)	
≥80	5(55.6)	4(44.4)	0.285*	7(77.8)	2(22.2)	0.113*
Sex						
Female	24(43.6)	31(56.4)		34(61.8)	21(38.2)	
Male	39(50.0)	39(50.0)	0.469	47(60.3)	31(39.7)	0.856
Location						
Colon	36(50.7)	35(49.3)		43(60.6)	28(39.4)	
Rectum	27(43.6)	35(56.5)	0.410	38(61.3)	24(38.7)	0.932
T stage						
T0-2	18(54.6)	15(45.5)		24(72.7)	9(27.3)	
T3-4	45(45.0)	55(55.0)	0.341	57(57.0)	43(43.0)	0.108
Lymph node						
No	31(47.7)	34(52.3)		42(64.6)	23(35.4)	
Yes	32(47.1)	36(52.9)	0.942	39(57.4)	29(42.7)	0.391
metastasis						
No	59(48.0)	64(52.0)		79(64.2)	44(35.8)	
Yes	4(40.0)	6(60.0)	0.628	2(20.0)	8(80.0)	0.006
Dukes’ stage						
A	14(56.0)	11(44.0)		19(76.0)	6(24.0)	
B	17(42.5)	23(57.5)		23(57.5)	17(42.5)	
C	28(48.3)	30(51.7)		37(63.8)	21(6.2)	
D	4(40.0)	6(60.0)	0.713*	2(20.0)	8(80.0)	0.019*
β-Catenin						
Negative	18(64.3)	10(35.7)		17(60.7)	11(39.3)	
positive	45(42.9)	60(57.1)	0.043	64(61.0)	41(39.1)	0.982
Chemotherapy						
No	40(48.2)	43(51.8)		49(59.0)	34(41.0)	
Yes	23(47.9)	25(52.1)	0.976	32,66.67	16(33.3)	0.384

There were 1 and 2 cases missing in location and chemotherapy, respectively.

* Trends p-value (Likelihood).

### Quantitative immunohistochemistry assays

Immunohistochemistry was done according to methods described previously [40], unstained tissue microarray sections were deparaffinized and hydrated in xylene and graded ethanol solutions, then placed in Ethylene Diamine Tetraacetic Acid (EDTA) antigenic repair buffer (pH9.0) in a microwave oven for antigenic repair. The slides were blocked with 3% H_2_O_2_ for 30 min, 3% BSA for 30 min, and then incubated overnight at 4°C with anti-*GDI1* primary antibody (1:1000, 66,434-1-ig, Proteintech, USA). The slides were probed with horseradish peroxidase-labeled polymer conjugated with corresponding antibodies for 50 min and DAB chromogenic program was conducted. Each slide was then counterstained with hematoxylin (GT100540, Gene).

We used the Aperio scanner (Aperio XT, LEICA), an automated imaging system, to obtain digital images of the stained sections for subsequent quantitative analyses. To reduce image reader bias, two independent investigators scored each sample in a double-blind manner. A joint review of investigators resolved discrepancies in scoring. Considering the heterogeneity of IHC staining, we displayed representative images of *GDI1* expression in the cytoplasm and membrane in Figure 3(a). The subcellular localization (cytoplasm, nucleus, membrane), stain intensity (integrated absorption), or percentage of stained cells (the total area or portion of cells positive) were scored for each image. A rating scale organized scores as such: negative (-), weak positive (+-), positive (+), and strong positive (++) (Figure 3(a)).

Based on the IHC staining distribution, we re-stratified cytoplasmic staining with ‘–’ or ‘+-’ as low-*GDI1* cytoplasm, cytoplasmic staining ‘+’ or ‘++’ as high- *GDI1* cytoplasm. For membrane staining, membranal staining ‘–’or ‘+-’ were regarded as low-*GDI1* membrane and membranal staining ‘+’ or ‘++’ as high-*GDI1* membrane.

### Statistical analyses

All statistical analyses were performed on the SAS statistical software, version 9.2 (SAS Institution Inc., Cary, NC). Student's t-test and one-way ANOVA were used for continuous data. The Pearson and Likelihood Chi-square test was used for categorical data. Kaplan–Meier plot was used to display the proportion of live (overall survival) or cancer-free (disease-free survival and relapse-free survival) patients by the length of follow-up in months. Hazard ratios (HR) with 95% confidence intervals (CI) were calculated using Cox proportional hazards regression analysis to examine the association of *GDI1* expression level with patient survival. Two-sided *P*-values of less than 0.05 were considered statistically significant.

The overall survival (OS) period was calculated as the time from initial surgery to the date when the patient was last seen. Only death from CRC was considered the end of the survival period. Disease-free survival (DFS) is defined as the time from initial surgery to tumor progression or relapse. All datasets were eligible for re-stratified *GDI1* and other related genes. To normalize the mRNA expression levels among the above datasets, we re-stratified *GDI1* scores into four levels (Q1, Q2, Q3, and Q4) based on the quartile value of each dataset. Values lower than the median were considered as low-*GDI1*, and values greater than or equal to median as high-*GDI1*. Cox analysis was performed on datasets using different and combined methods.

## Results

To address our hypothesis that *GDI1* may play essential roles in the aggressiveness of CRC, we investigated the relationships between mRNA expression of *GDI1* and differentiation, proliferation, and invasion of CRC in six gene expression datasets, and the results indicated that *GDI1* significantly enhanced the aggressiveness of CRC. Furthermore, outcome analyseis were performed to consistently demonstrated if mRNA expression of that *GDI1* impacts was dramatically associated with poor survival of CRC patients. The prognostic meaning of GDI1 protein expression in cytoplasm and membrane was also validated in our collected CRC cohort by using IHC analysis. CRC patients in stage III whose GDI1 expression was low with chemotherapy have a high OS. Based on these findings, *GDI1* may act as a potential prognostic biomarker in treating patients with CRC.

### GDI1 *expression is associated with the clinical features of colorectal cancer*

**Figure 1.**
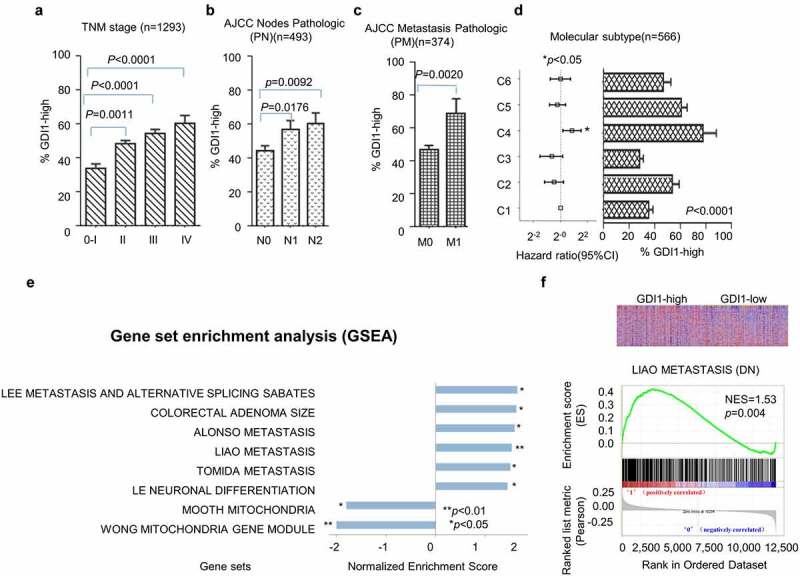
Distribution of the GDI1 mRNA expression and aggressiveness of CRC

The association between *GDI1* expression and clinical features was assessed on downloaded human tissue gene expression datasets. Univariate analysis revealed that the expression of *GDI1* was significantly related to age, tumor node metastasis (TNM) stage, and molecular subtype (Table 1 and Figure 1(a,d)) (*p* < 0.05). Meanwhile, a Cox proportional hazard model further validated that age (≥50 yrs), advanced TNM stage, bigger tumor, lymph node involvement, metastasis, poor differentiation, and molecular subtype (C4) as the vital risk factors for poor prognosis (*p* < 0.05) (Table 1). Interestingly, GDI1 was overexpressed in the C4 molecular subtype of CRC, which has the poorest outcome (HR = 1.79, 95% 1.12–2.84, C4 *vs* C1, *p* = 0.02) (Figure 1(d)). The results suggested that overexpression of *GDI1* is associated with poor differentiation and aggressiveness of CRC.

Patients’ gene expression profiles were downloaded. a) The mRNA expression of *GDI1* and AJCC TNM stages of CRC. b) *GDI1* expression and lymph node involvement. c) *GDI1* and distant metastasis. d) The left panel is the HR of each molecular subtype (vs. C1 subgroup). The right panel is the distribution of *GDI1* in each molecular subgroup. e) Gene set enrichment analysis (GSEA) was performed to search *GDI1*-enriched gene signatures and signaling pathways in the GSE39582 dataset. *GDI1* enriched cancer-related gene signatures are listed. f) The *GDI1*-enriched Liao metastasis signature was displayed as representative results of GSEA. In the upper panel, all genes were sorted out based on the rank between high-*GDI1*/ low-*GDI1*. The enrichment score was calculated based on the score of Liao metastasis signature genes. The heatmap is displayed on the lower panel. Each column represents one tissue sample, and each row represents each gene of Liao metastasis signature. Red means upregulated, and blue means downregulated. Most Liao metastasis signatures were upregulated in high-*GDI1* subgroup but downregulated in the low-*GDI1* subgroup in heatmap.

The representative results of the GSE39582 set analysis are shown in Figure 1(e). Six high-*GDI1*-enriched six gene signatures included Lee metastasis and alternative splicing Sabates, Colorectal adenoma size, Alonso Metastasis, Liao Metastasis, Tomida Metastasis, and Le Neuronal differentiation. Meanwhile, low-*GDI1* was enriched in signatures of Mooth Mitochondria and Wong Mitochondria gene module. Figure 1(f) shows the *GDI1*-enriched LIAO METASTASIS. The normalized enrichment score (NES) was 1.53 (*p* = 0.004). Furthermore, the protein–protein interaction network of GDI1 was obtained from STRIG.ORG (S. Figure 1). These proteins were involved in cancer invasion (members of RAS oncogene family and Ras homolog family members), cell cycle regulation, neoplastic processes and inflammation (PAK2) [41,42], cellular proliferation, polarity, adhesion, and migration (CDC42) [43], proliferation, signaling, secretion, cytoskeletal organization and proliferation (ARHGDIA and ARHGDIB) [44] and others. Most of these proteins were related to cancer cell proliferation and invasion. The mRNA and protein expression levels of *GDI1* might be related to the aggressiveness of CRC.

### GDI1 *is a potential prognostic factor in colorectal cancer.*

**Figure 2.**
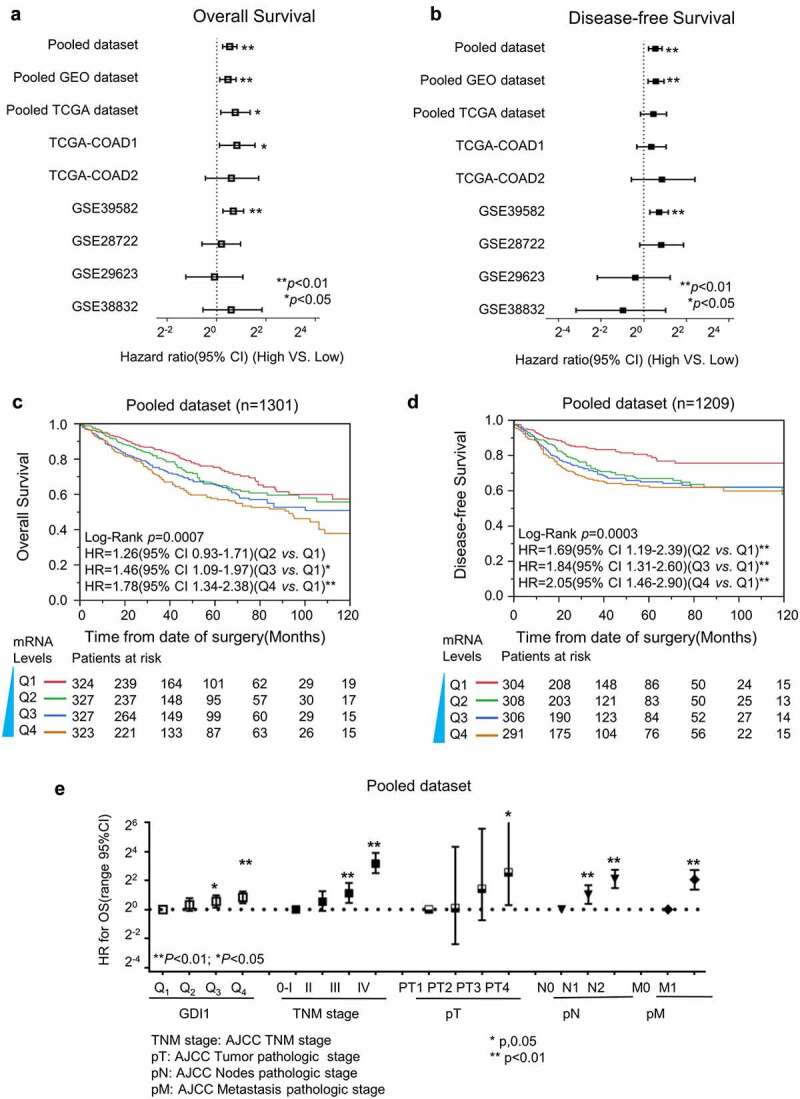
Survival analysis for *GDI1* mRNA expression and outcome of CRC

We first used Cox proportional hazard analysis to estimate the HR of *GDI1* in each CRC dataset. A meta-analysis was then employed to assess the overall prognostic value of *GDI1* mRNA in these datasets (Figure 2(a,b)). It was suggested that high expression of *GDI1* mRNA was significantly associated with a high relative risk of death and recurrence in CRC patients.

For each dataset, the HR of *GDI1* was estimated by univariate Cox proportional hazard analysis. Then, the overall HR of *GDI1* was summarized by meta-analysis. Meanwhile, all participants from different datasets were merged after quantile normalization. The Kaplan–Meier plot visualized the *GDI1* expression levels and outcomes of CRC. a) Meta-analysis of *GDI1* and OS of CRC. b) Meta-analysis of DFS for CRC. c) Pooled Kaplan–Meier analysis for *GDI1* and OS of CRC. d) Pooled Kaplan–Meier analysis for *GDI1* and DFS of CRC. e) Parallel comparison of the prognostic performance of *GDI1* and TNM stages. The HRs were based on Cox analysis in the GSE39582 dataset.

Survival analysis was conducted for each dataset using uni- and multi-covariate Cox proportional hazard analysis, and participants in each dataset were re-categorized into four subgroups (Q1, Q2, Q3, and Q4) according to the expression levels of *GDI1*. The results are listed in Table 2. Q1, the subgroup with the lowest expression, was set as the relative point of reference. Mostly, the HR of *GDI1* for OS and PFS increased with its mRNA level. In the groups with higher *GDI1* levels (e.g., Q4), the association was more significant in almost all datasets. The overall pooled analysis demonstrated that the HR of higher *GDI1* (Q4) was 1.78 [95% confidence interval (CI): 1.34–2.38] for OS and 2.05 (95% CI: 1.46–2.90) for PFS.

Figure 2(c,d)) illustrates that the mRNA level of *GDI1* is positively related to the OS and DFS of CRC patients. Like TNM stage, *GDI1* level increases with the relative risk of death (Figure 2(e)).

### *Prognostic significance of* GDI1 *is validated in CRC human subjects*

**Figure 3.**
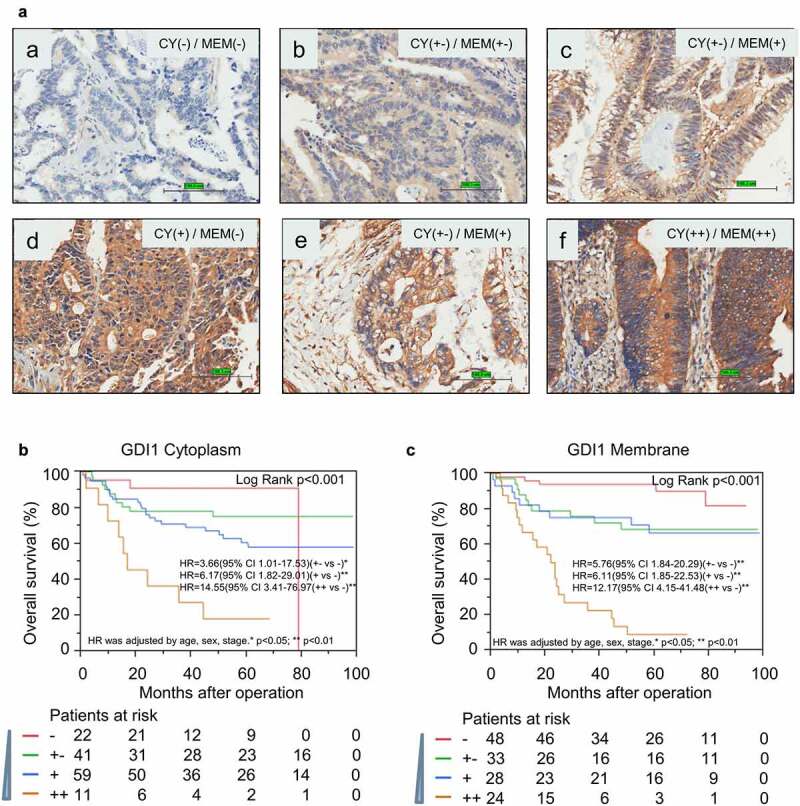
Immunohistochemistry analysis for the protein expression of GDI1 in the cytoplasm and membrane

Immunohistochemistry analysis was performed on formalin fixed paraffin-embedded (FFPE) CRC tissues. We used the well-specificity *GDI1* antibody, whose condition was optimized on a colorectal tissue assay. Since GDI1 is a secreted protein, the signals of IHC staining in the membrane and cytoplasm were scored separately. Representative IHC results of GDI1 are shown in Figure 3(a).

The GDI1 antibody condition was optimized on a CRC board, and the specificity of the GDI1 antibody was also validated. a) The expression of GDI1 was scored by signal density in the cytoplasm and membrane. The representative score of GDI1 was displayed from (a)-(f). (a) Both cytoplasmic and membrane negative staining in CRC cells; (b) Cytoplasm weak positive and membrane weak positive staining; (c) Cytoplasm weak positive and membrane positive staining; (d) Cytoplasm positive and membrane negative staining; (e) Cytoplasm weak positive and membrane positive staining; (f) Strong positive in both cytoplasm and membrane. (b) Kaplan–Meier curve was plotted for *GDI1* cytoplasm score and OS. Cox proportional hazard model determined the hazard ratio (HR). HR was adjusted by age, sex, Dukes’ stage. * p < 0.05; ** p < 0.01. c) Survival analysis for *GDI1* membrane score and outcome of CRC.

As shown in Table 3, the protein level of membrane GDI1 was significantly linked to distant metastasis and Dukes’ stage (*p* < 0.05). The protein level of cytoplasmic GDI1 was only related to β-Catenin positive expression (*p* < 0.05). Survival analysis further validated that protein levels of GDI1 in both cytoplasm and membrane were significantly associated with the outcome of CRC in the ZJU set (Figure 3(b,c)). A higher expression of GDI1 protein was related to a worse outcome of CRC, which is compatible with findings from *GDI1* mRNA analysis.

### *Overexpression of* GDI1 *may be related to chemoresistance against CRC*

**Figure 4.**
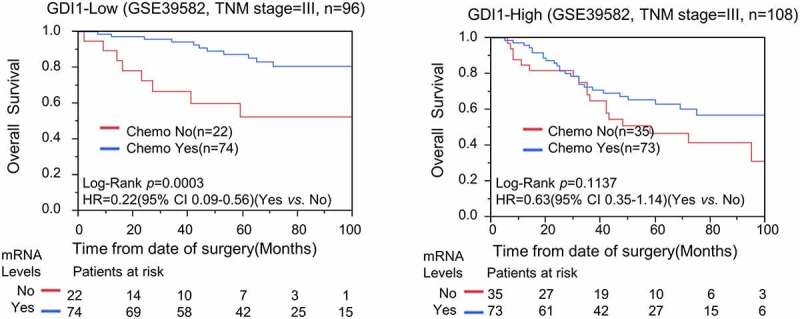
Stratification analysis for *GDI1* expression and chemotheresistance in CRC patients

Chemotherapy is generally administered to patients with advanced stages of CRC. Here, we address whether the expression level of *GDI1* is associated with the sensitivity of chemotherapy to CRC. To avoid the confounding effects of TNM stage, we only analyzed chemoresistance in patients with stage-III CRC. Kaplan–Meier and Cox analyses demonstrated that chemotherapy prolonged OS significantly in low-*GDI1* subgroup (log-rank *p* = 0.0003; HR = 0.22; 95% CI: 0.09–0.56), but not in the high-*GDI1* subgroup (log-rank *p* = 0.1137) (Figure 4(a,b)). Hence, our findings suggest that overexpression of *GDI1* may be associated with chemoresistance against CRC.

We only selected stage-III CRC patients in the GSE39582 dataset to reduce the confounding effects. The participants were stratified into High-*GDI1* and low-*GDI* subgroups. The efficacy of chemotherapy was evaluated by Kaplan–Meier and Cox analyses. a) Chemotherapy significantly reduced the relative risk of death in low-*GDI1* group. b) Chemotherapy did not reduce the relative risk of death in high-*GDI1* group.

## Discussion

CRC is a common malignant tumor of the digestive system and a leading cause of cancer-related death worldwide [45]. Despite the new treatment efforts, its prognosis is still poor, mainly due to the high rate of distant metastasis [46,47]. Therefore, effective biomarkers are in urgent need to improve the treatments of CRC. In the present study, we for the first time reported the prognostic value of *GDI1* for CRC outcomes. We discovered the relationship of *GDI1* with clinical factors and outcomes of CRC. The expression of *GDI1* was associated with the stage of CRC (Table 2 and Figure 1). In particular, *GDI1* expression was significantly related to CRC metastasis in Cox regression analyses and GSEA (Table 2 and Figure 1). Kaplan–Meier analysis confirmed that *GDI1* expression had a negative impact on the survival of CRC patients (Figure 2). Furthermore, the prognostic significance of *GDI1* was validated using tissue samples. As *GDI1* protein expression increased, the relative risk of death increased (Figure 3). Heterogeneity and localization of GDI1 protein were also taken into consideration. Both cytoplasmic and membranal expressions of *GDI1* were significantly related to the poor prognosis of CRC.

Our study is also more innovative and interesting in its subgroup analysis on the association between *GDI1* expression and chemoresistance, compared to the simple analysis of the prognostic role of a certain gene [37]. Our preliminary analysis demonstrated that chemotherapy could significantly reduce the risk of death in stage-III CRC patients with low-*GDI1*, but not in those with *GDI1*-high (Figure 4). Potentially, *GDI1* might serve as a predictive biomarker for stage III CRC. Meanwhile, it was implied that suppression of *GDI1* might enhance the chemosensitivity of CRC.

One limitation of our study is that assays *in vitro* were not conducted to investigate whether *GDI1* inhibition may decrease CRC cells’ proliferative and invasive ability. Due to insufficient CRC patients with stage III, the relationship between *GDI1* expression and chemosensitivity could not be validated in a cohort. In addition, the mechanism of *GDI1* in CRC progression and chemoresistance should be explored with more animal and clinical studies.

## Conclusion

Overexpression of *GDI1* is associated with the aggressiveness and poor outcomes of CRC. *GDI1* can be used as a biomarker to predict CRC prognosis and design new treatment options.

## Supplementary Material

Supplemental Material

## Abbreviations

95% CI, 95% confidence interval; GSEA, gene set enrichment analysis; OS, overall survival; DFS, disease-free survival; CRC, colorectal cancer; HR, hazard ratio; IHC, immunohistochemistry; TNM, tumor node metastasis; ZJU, Zhejiang University; GDI, GDP dissociation inhibitor.
