# Supplementary material for: Overexpression of GDP dissociation inhibitor 1 gene associates with the invasiveness and poor outcomes of colorectal cancer
Source: Bioengineered. 2021 Sep 13;12(1):5595–606. doi: 10.1080/21655979.2021.1967031 (PMC8806759; doi:10.1080/21655979.2021.1967031)
Supplement: Supplemental Material [file KBIE_A_1967031_SM3381.zip › suppl/suppl tab.pdf]

Supplementary Table 1: Overall review of published microarray data sets

**Data set**	**GSE39582**	**GSE38832**	**GSE28722**	**GSE29623**	**TCGA-COAD1**	**TCGA-COAD2**
No.of patients	585	122	250	130	278	151
Assessable cases*	566	122	125	65	278	151
Date of study	1997-2007	N/A	N/A	N/A	1998-2013	2003-2010
Microarray	Affimetrix HG-U133	Affimetrix HG-U133	Rosetta custom human 23K	Affimetrix HG-U133		
Platforms*	GPL570	GPL570	GPL13425	GPL570		
Country	France	USA	USA	USA		
GDI1 probes	201864_at	201864_at	Y	201864_at	Y	Y
Age at diagnosis	68 (22-97)	N/A	67 (21-91)	N/A	67 (31-90)	72 (36-90)
Sex	Y	Y	Y	Y	Y	Y
Elson grade	N/A	N/A	N/A	Y	N/A	N/A
Tumor size	N/A	N/A	N/A	N/A	N/A	N/A
Lymph node	N/A	N/A	N/A	N/A	Y	Y
Metastasis	N/A	N/A	Y	N/A	Y	Y
AJCC stage	Y	Y	Y	Y	Y	Y
Molecular subtype	Y	N/A	N/A	N/A	N/A	N/A
Chemotherapy	Y	N/A	N/A	Y	N/A	N/A
Radiotherapy	N/A	N/A	N/A	N/A	N/A	N/A
Hormone therapy	N/A	N/A	N/A	N/A	N/A	N/A
OS months† (Range)	N/A	0.3-111.4	0.1-14.3	1.4-120.6	0-147.9	0-54.0
DFS months‡ (Range)	0-201.0	0.3-111.4	0.1-14.3	1.4-105.2	0-147.9	0-54.0

* Patients without clinical information, follow-up data or *GDI1* expression level were excluded from this study.

†OS: Overall survival; ‡ DFS: Progression-free survival/Relapse-free survival
