# Supplementary material for: Overexpression of GDP dissociation inhibitor 1 gene associates with the invasiveness and poor outcomes of colorectal cancer
Source: Bioengineered. 2021 Sep 13;12(1):5595–606. doi: 10.1080/21655979.2021.1967031 (PMC8806759; doi:10.1080/21655979.2021.1967031)
Supplement: Supplemental Material [file KBIE_A_1967031_SM3381.zip › suppl/supplifiglegend.pdf]

Figure 3. Immunohistochemistry analysis for the protein expression of GDI1 in the cytoplasm and membrane. The GDI1 antibody condition was optimized on a CRC board, and the specificity of the GDI1 antibody was also validated. A) The expression of GDI1 was scored by signal density in the cytoplasm and membrane. The representative score of GDI1 was displayed from (a)-(f). (a) Both cytoplasmic and membrane negative staining in CRC cells; (b) Cytoplasm weak positive and membrane weak positive staining; (c) Cytoplasm weak positive and membrane positive staining; (d) Cytoplasm positive and membrane negative staining; (e) Cytoplasm weak positive and membrane positive staining; (f) Strong positive in both cytoplasm and membrane. (B) Kaplan-Meier curve was plotted for *GDI1* cytoplasm score and OS. Cox proportional hazard model determined the hazard ratio (HR). HR was adjusted by age, sex, Dukes' stage. * $p<0.05$; ** $p<0.01$. C) Survival analysis for *GDI1* membrane score and outcome of CRC.

Figure 4. Stratification analysis for *GDI1* expression and chemotheresistance in CRC patients. We only selected stage-III CRC patients in the GSE39582 dataset to reduce the confounding effects. The participants were stratified into High-*GDI1* and low-*GDI* subgroups. The efficacy of chemotherapy was evaluated by Kaplan-Meier and Cox analyses. A) Chemotherapy significantly reduced the relative risk of death in low-*GDI1* group. B) Chemotherapy did not reduce the relative risk of death in high-*GDI1* group.

Supplementary Figure legend

Supplementary Figure 1: Protein-protein interaction analysis for GDI1. The protein interaction network of GDI1 was predicted on the STRING website.
